# Prompt rewetting of drained peatlands reduces climate warming despite methane emissions

**DOI:** 10.1038/s41467-020-15499-z

**Published:** 2020-04-02

**Authors:** Anke Günther, Alexandra Barthelmes, Vytas Huth, Hans Joosten, Gerald Jurasinski, Franziska Koebsch, John Couwenberg

**Affiliations:** 1 University of Rostock, Faculty of Agricultural and Environmental Studies, Landscape Ecology, Rostock, Germany; 2 University of Greifswald, Faculty of Mathematics and Natural Sciences, Peatland Studies and Paleoecology, Greifswald, Germany; 3 Greifswald Mire Centre (GMC), Greifswald, Germany

**Keywords:** Climate-change ecology, Environmental impact

## Abstract

Peatlands are strategic areas for climate change mitigation because of their matchless carbon stocks. Drained peatlands release this carbon to the atmosphere as carbon dioxide (CO_2_). Peatland rewetting effectively stops these CO_2_ emissions, but also re-establishes the emission of methane (CH_4_). Essentially, management must choose between CO_2_ emissions from drained, or CH_4_ emissions from rewetted, peatland. This choice must consider radiative effects and atmospheric lifetimes of both gases, with CO_2_ being a weak but persistent, and CH_4_ a strong but short-lived, greenhouse gas. The resulting climatic effects are, thus, strongly time-dependent. We used a radiative forcing model to compare forcing dynamics of global scenarios for future peatland management using areal data from the Global Peatland Database. Our results show that CH_4_ radiative forcing does not undermine the climate change mitigation potential of peatland rewetting. Instead, postponing rewetting increases the long-term warming effect through continued CO_2_ emissions.

## Abstract (web summary)

Drained peatlands are sources of CO_2_, and though rewetting could curb emissions, this strategy results in elevated methane release. Here, the authors model peatland emissions scenarios and show that rewetting is a critical way to mitigate climate change despite potential methane increases.

## Introduction

Each year, drained peatlands worldwide emit ~2 Gt carbon dioxide (CO_2_) by microbial peat oxidation or peat fires, causing ~5% of all anthropogenic greenhouse gas (GHG) emissions on only 0.3% of the global land surface^1^. A recent study states that the effect of emissions from drained peatlands in the period 2020–2100 may comprise 12–41% of the remaining GHG emission budget for keeping global warming below +1.5 to +2 °C^2^. Peatland rewetting has been identified as a cost-effective measure to curb emissions^3^, but re-establishes the emission of methane (CH_4_). In light of the strong and not yet completely understood impact of CH_4_ on global warming^4,5^ it may seem imprudent to knowingly create or restore an additional source. Furthermore, there is considerable uncertainty on emissions from rewetted peatlands and some studies have reported elevated emissions of CH_4_ compared with pristine peatlands^6–9^.

The trade-off between CH_4_ emissions with and CO_2_ emissions without rewetting is, however, not straightforward: CH_4_ has a much larger radiative efficiency than CO_2_^10^. Yet, the huge differences in atmospheric lifetime lead to strongly time-dependent climatic effects. Radiative forcing of long-term GHGs (in case of peatlands: CO_2_ and N_2_O) is determined by cumulative emissions, because they factually accumulate in the atmosphere. In contrast, radiative forcing of near-term climate forcers (in case of peatlands: CH_4_) depends on the contemporary emission rate multiplied with the atmospheric lifetime^10,11^, because resulting atmospheric concentrations quickly reach a steady state of (sustained) emission and decay. Meanwhile, common metrics like global warming potential (GWP) and its sustained flux variants^11,12^ fail to account for temporal forcing dynamics. These different atmospheric dynamics are relevant for the question how the various management scenarios will influence global climate and whether a scenario will amplify or attenuate peak global warming, i.e., the maximum deviation in global surface temperatures relative to pre-industrial times. An amplification of peak warming increases the risk of reaching major tipping points in the Earth’s climate system^13,14^.

Here, we explore how the different lifetimes of CO_2_/N_2_O vs. CH_4_ play out when assessing options for peatland rewetting as a climate warming mitigation practice by comparing five global scenarios (Table 1). These scenarios represent extreme management options and exemplify the differences caused by timing and extent of rewetting. For our modeling exercise, we focus on the direct human-induced climatic effects and conservatively assume pristine peatlands to be climate-neutral. Further, we assume that the maximum peatland area to be drained during the 21st century equals the area that is already drained in 2018 (505,680 km², Global Peatland Database^15^) plus an additional ~5000 km² per year (average net increase of drained peatland area between 1990 and 2017^16^). For all scenarios, we apply IPCC default emissions factors^17^ as sustained fluxes. To compare the radiative forcing effects of the different GHGs, we use a simplified atmospheric perturbation model that has been shown to provide reliable estimates of the climatic effects of peatlands^18^ (see Methods). Our results show that total radiative forcing quickly reaches a plateau after rewetting, because of the halted emissions of CO_2_/N_2_O of rewetted peatlands and the short atmospheric lifetime of any emitted CH_4_. In contrast, postponing rewetting has a long-term warming effect resulting from continued CO_2_ emissions. Warnings against CH_4_ emissions from rewetted peatlands are therefore unjustified in the context of effective climate change mitigation.

**Table 1:** Global scenarios of peatland management.

Scenario	Description
Drain_More	The area of drained peatland continues to increase from 2020 to 2100 at the same rate as between 1990 and 2017
No_Change	The area of drained peatland remains at the 2018 level
Rewet_All_Now	All drained peatlands are rewetted in the period 2020–2040
Rewet_Half_Now	Half of all drained peatlands are rewetted in the period 2020–2040
Rewet_All_Later	All drained peatlands are rewetted in the period 2050–2070

## Results and Discussion

### Radiative forcing dynamics of global scenarios

**Fig. 1:**
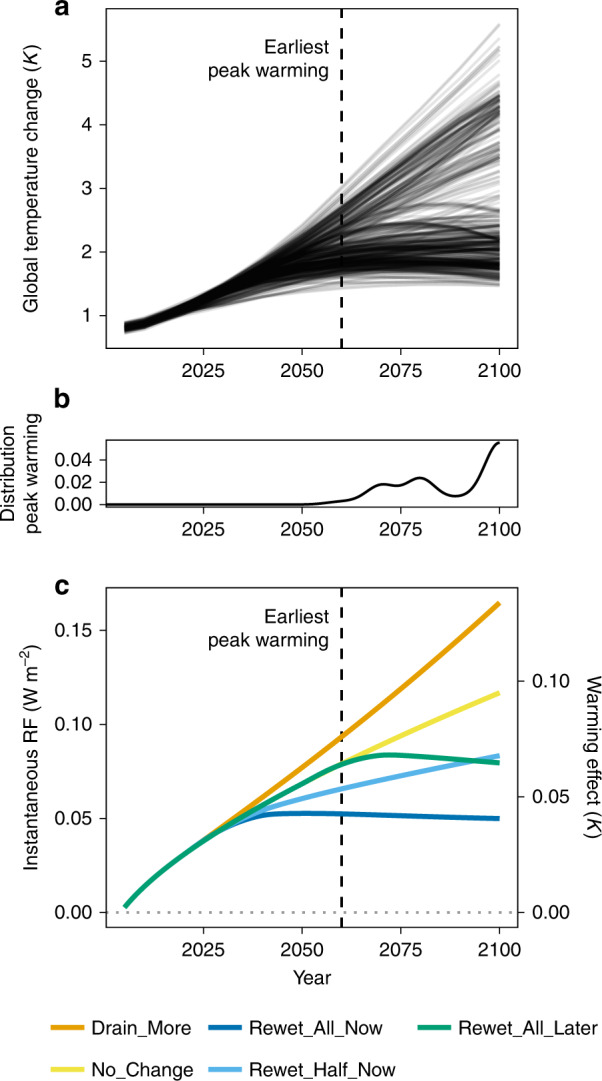
Global warming and climatic effects of peatland management. Mean global temperature change relative to 2005 (**a**) and frequency distribution of the timing of peak warming (**b**) according to AR5 model pathways (downloaded from IAMC AR5 Scenario Database) are shown compared with radiative forcings (RF) and estimated instantaneous warming effects of global peatland management scenarios (panel **c**, own calculations). Please note that in panel **c**) forcing of peatlands that remain pristine is assumed to be zero.

Rewetting of drained peatlands instantly leads to climatic benefits compared with keeping the status quo (Fig. 1). In case of rewetting all drained peatlands (scenarios Rewet_All_Now and Rewet_All_Later, see Table 1) the radiative forcing stops increasing followed by a slow decrease. Since the response of global temperature is lagging behind changes in total radiative forcing by 15–20 years^19^, peatlands should be rewetted as soon as possible to have most beneficial (cooling) effects during peak warming, which AR5 climate models expect to occur after ~2060 with increasing probability towards the end of the century (Fig. 1).

**Fig. 2:**
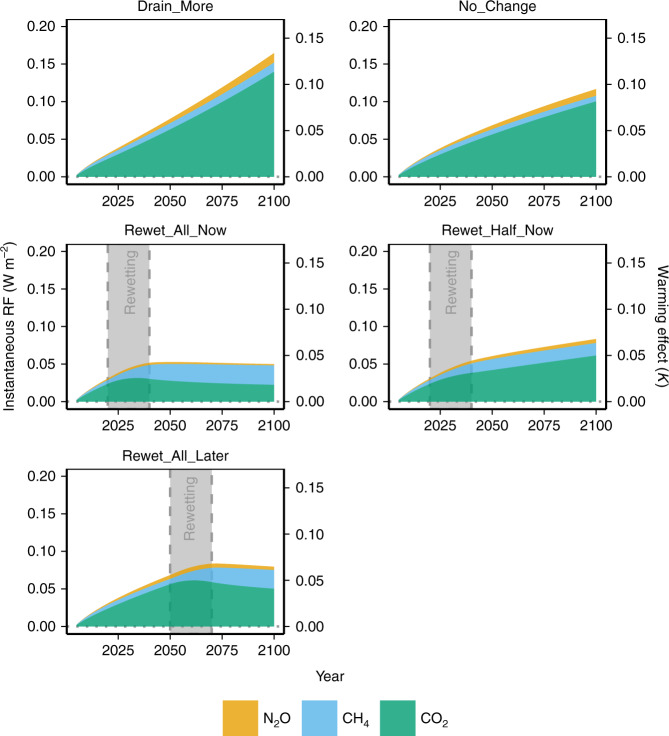
Climatic effects of peatland scenarios by greenhouse gas. Contributions of the different greenhouse gases (nitrous oxide, N_2_O, methane, CH_4_, and carbon dioxide, CO_2_) to total radiative forcing (RF) are shown with estimated warming effects in the modeled scenarios. The gray area shows the period of rewetting. Note that in the figure forcing of peatlands that remain pristine is assumed to be zero.

**Fig. 3:**
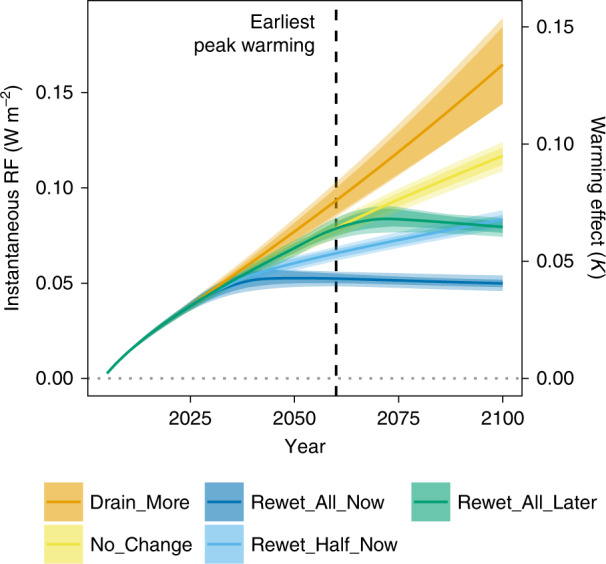
Modeling sensitivity to variation of input values. The influence of modeling choices and uncertainty of emission factors on radiative forcing (RF) and on estimated warming effects is shown for the five global peatland scenarios. Error ranges represent the range (minimum to maximum) of radiative forcing resulting from random variations in ongoing drainage rate (1000–8000 km² per year) and emission factors (10 and 20% uncertainty of emission factor, represented by shading intensity).

The overall climatic effect of peatland rewetting is indeed strongly determined by the radiative forcing of sustained CH_4_ emissions (Fig. 2). However, because of the negligible or even negative emissions of CO_2_/N_2_O of rewetted peatlands and the short atmospheric lifetime of CH_4_, the total anthropogenic radiative forcing of all three GHGs combined quickly reaches a plateau after rewetting. Meanwhile, differences in radiative forcing between drainage (increased forcing) and rewetting scenarios (stable forcing) are mainly determined by differences in the forcing of CO_2_ (Fig. 2). Rewetting only half of the currently drained peatlands (Rewetting_Half_Now) is not sufficient to stabilize radiative forcing. Instead, CO_2_ from not-rewetted peatland keeps accumulating in the atmosphere and warming the climate. Note that in the Rewet_Half_Now scenario CH_4_ forcing is more than half that of the Rewet_All_… scenarios, because drained peatlands also emit CH_4_, most notably from drainage ditches. Comparing the scenarios Rewet_All_Now and Rewet_All_Later shows that timing of peatland rewetting is not only important in relation to peak temperature, but also with respect to the total accumulated CO_2_ and N_2_O emissions in the atmosphere and the resulting radiative forcing (Fig. 2). These patterns are valid also when considering possible future changes of new drainage rate or emission factors (Fig. 3).

### General conclusions for global peatland management

Our simulations highlight three general conclusions: First, the baseline or reference against which peatland rewetting has to be assessed is the drained state with its large CO_2_ emissions. For this reason, rewetted peatlands that are found to emit more CH_4_ than pristine ones^9^ are no argument against rewetting. Moreover, whereas rewetted peatlands may again become CO_2_ sinks, the faster and larger climatic benefits of peatland rewetting result from the avoidance of CO_2_ emissions from drained peatlands. Second, the climate effect is strongly dependent on the concrete point in time that rewetting is implemented. This fact is hitherto insufficiently recognized because it remains hidden by the common use of metrics that involve predetermined time horizons (like GWP or sustained flux variants of GWP). Finally, in order to reach climate-neutrality in 2050 (as implied by the Paris Agreement on limiting the increase in global temperature to well below 2 °C), it is insufficient to focus rewetting efforts on selected peatlands only: to reach the Paris goal, CO_2_ emissions from (almost) all drained peatlands have to be stopped by rewetting^2^.

Limiting global warming requires immediate reduction of global GHG emissions. It has been suggested that the negative climate effects of drained peatlands could be offset by growing highly-productive bioenergy crops^20^ or wood biomass^21^ as substitute for fossil fuels. In this study, we did not include this option because similar biomass-based substitution benefits can also be reached by cultivating biomass on rewetted peatlands^22^, i.e., without CO_2_ emissions from drained peat soil.

In conclusion, without rewetting the world’s drained peatlands will continue to emit CO_2_, with direct negative effects on the magnitude and timing of global warming. These effects include a higher risk of reaching tipping points in the global climate system and possible cascading effects^13^. In contrast, we show that peatland rewetting can be one important measure to reduce climate change and attenuate peak global warming: The sooner drained peatlands are rewetted, the better it is for the climate. Although the CH_4_ cost of rewetting may temporarily be substantial, the CO_2_ cost of inaction will be much higher.

## Methods

### Scenarios

Drained peatland area was taken from the Global Peatland Database (GPD)^15^, which includes, among other things, national data from the most recent UNFCCC National Inventory Submissions and Nationally Determined Contributions. We used data separated by IPCC climate zone (boreal, temperate, and tropical) and assigned land use categories. Available land use categories were Forest, Cropland, Deep-drained grassland, Shallow-drained grassland, Agriculture (i.e., either grassland or cropland when the original data source did not differentiate between these two categories), and Peat extraction (see Table 2). Because of their only small area and uncertain emission factors, arctic drained peatlands (~100 kha) were neglected. The average net increase of drained peatland area between 1990 and 2017^16^ assumed for the Drain_More scenario includes the disappearance of drained peatlands that have lost all their peat deposits. Newly drained/rewetted area in the scenarios is distributed across the climatic zones (and land use classes) according to the relative proportions of today’s drained peatland area. As future drainage—similar to the past two decades^16^—will probably focus on tropical and subtropical peatlands, our Drain_More scenario likely underestimates the climate effects of future drainage. For information on how variations in the assumed drainage rate and uncertainty of emission factors affected the displayed radiative forcing effects of the scenarios please see Fig. 3.

**Table 2:** Areas of drained peatland (kha) by climate zone and land use category according to the Global Peatland Database, together with aggregated emission factors.

Climatic zone	Land use category	Area (kha)	CO_2_ (t ha^−1^ a^−1^)	CH_4_ (kg ha^−1^ a^−1^)	N_2_O (kg ha^−1^ a^−1^)
Boreal	Forest	5474	2.5	9.8	2.6
	Cropland	262	27.9	58.3	19.4
	Deep-drained grassland	426	20.2	59.6	14.2
	Shallow-drained grassland	0	—	—	—
	Agriculture	3420	24.1	43.0	16.8
	Peat extraction	333	10.2	32.9	0.5
	Rewetted	—	−1.3	123.6	0
Temperate	Forest	6315	10.3	7.9	4.3
	Cropland	2528	28.6	58.3	19.4
	Deep-drained grassland	3405	22.3	73.5	12.3
	Shallow-drained grassland	2422	13.6	63.4	2.4
	Agriculture	8389	21.0	55.8	10.1
	Peat extraction	662	10.8	32.9	0.5
	Rewetted	—	−0.4	205.9	0
Tropical	Forest	7235	22.0	50.0	3.7
	Cropland	305	45.0	118.9	4.2
	Deep-drained grassland	70	37.4	52.0	7.7
	Shallow-drained grassland	0	—	—	—
	Agriculture	9314	42.5	96.6	5.4
	Peat extraction	8	10.1	32.9	5.6
	Rewetted	—	1.9	166.5	0

Emission factors assumed for rewetted peatlands are also shown for each climatic zone.

### Emissions

Emission factors for each climate zone and land use category were taken from the IPCC Wetland supplement^17^ that currently presents the most robust and complete meta-study of published emission data. We applied all emission factors as sustained fluxes. Emission factors were averaged for IPCC categories that were given at a higher level of detail (e.g., nutrient-poor vs. nutrient-rich boreal forest) than the available land use categories from the GPD. Equally, we averaged the supplied emission factors for grassland and cropland in order to obtain emission factors of the land use class Agriculture (see Table 2 for final aggregated emission factors and Supplementary Table 1 for exact aggregation steps). We included emissions from ditches and DOC exports by using emission factors and default cover fraction of ditches given by the IPCC^17^ (Supplementary Table 1). Since the IPCC Wetlands Supplement does not provide an emission factor for CH_4_ from tropical peat extraction sites, we assumed the same CH_4_ emissions as for temperate/boreal peat extraction. Values of the emission factors could change slightly when more emission data becomes available. To cover this possibility, we randomly varied all emission factors within a range of 10–20% uncertainty in our sensitivity analysis (Fig. 3). Please note that these uncertainty ranges do not correspond to the confidence intervals given by the IPCC Wetlands Supplement, which describe the observed variability of emissions from individual peatlands. Since our analyses take a global perspective, our sensitivity analyses instead cover possible changes of the mean emissions (i.e., emission factors). In addition, individual studies have discussed the presence of a CH_4_ peak for the first years after rewetting^7,8^. Although this is likely not a global phenomenon^23^, please see Supplementary Fig. 1 for an estimate of the uncertainty related to possible CH_4_ peaks.

### Radiative forcing

The forcing model uses simple impulse-response functions^24^ to estimate radiative forcing effects of atmospheric perturbations of CO_2_, CH_4_, and N_2_O fluxes^12^. Perturbations of CH_4_ and N_2_O were modeled as simple exponential decays, while CO_2_ equilibrates with a total of five different pools at differing speeds. For CO_2_, we adopted the flux fractions and perturbation lifetimes used by ref. ^18^. In the model, we assume a perfectly mixed atmosphere without any feedback mechanisms but include indirect effects of CH_4_ on other reagents^10^.

Climatic effects of CO_2_ from CH_4_ oxidation should not be considered for CH_4_ from biogenic sources^10^. However, although the large majority of CH_4_ from peatlands stems from recent plant material (a biogenic source), the proportion of fossil CH_4_ (from old peat) may be substantial in some cases^25^. Thus, we conservatively included the climatic effect of CO_2_ from CH_4_ oxidation in our analyses. Overall, this forcing comprised only 5–7% of the CH_4_ radiative forcing and only ~1–3% of total radiative forcing.

We compare the radiative forcing trajectories of the various peatland management scenarios with the global temperature change as projected by all available pathways of IPCC’s AR5 and use the same starting year 2005 as these pathways. Further, we estimated the approximate effects of radiative forcing on global mean temperature as ~1 K per 1.23 W/m² radiative forcing^26^.

### Supplementary information

Supplementary Figure 1 and Supplementary Table 1

Supplementary Code

Peer Review File

## Data Availability

The models for projected temperature change were downloaded from IAMC AR5 Scenario Database (available at https://secure.iiasa.ac.at/web-apps/ene/AR5DB). Emission factors and peatland cover data are entirely included in the paper.

## Code availability

The code for the atmospheric perturbation model can be found in the supplementary information. Code was written by A. Günther in the R programming language.
