# Author Correction: Prompt rewetting of drained peatlands reduces climate warming despite methane emissions

**DOI:** 10.1038/s41467-024-47604-x

**Published:** 2024-04-12

**Authors:** Anke Günther, Alexandra Barthelmes, Vytas Huth, Hans Joosten, Gerald Jurasinski, Franziska Koebsch, John Couwenberg

**Affiliations:** 1 University of Rostock, Faculty of Agricultural and Environmental Studies, Landscape Ecology, Rostock, Germany; 2 University of Greifswald, Faculty of Mathematics and Natural Sciences, Peatland Studies and Paleoecology, Greifswald, Germany; 3 Greifswald Mire Centre (GMC), Greifswald, Germany

Correction to: *Nature Communications* 10.1038/s41467-020-15499-z, published online 02 April 2020

The original version of the Supplementary Code associated with this Article contained an error on p. 5, lines 163–165, which incorrectly read

‘## Convert all inventories to kg,

## then calculate individual instantaneous radiative forcing

dataf$n2orf ← (n2oinv/1000/14 *44)*radeff.n2o*ie.n2o’

The correct version states ‘dataf$n2orf ← (n2oinv/1000/28*44)*radeff.n2o*ie.n2o’ in place of ‘dataf$n2orf ← (n2oinv/1000/14 *44)*radeff.n2o*ie.n2o’.

The HTML has been updated to include a corrected version of the Supplementary Code.

### Supplementary information

Updated Supplementary Code

