# Supplementary material for: Prompt rewetting of drained peatlands reduces climate warming despite methane emissions
Source: Nat Commun. 2020 Apr 2;11:1644. doi: 10.1038/s41467-020-15499-z (PMC7118086; doi:10.1038/s41467-020-15499-z)
Supplement: Supplementary file 1 — Supplementary Figure 1 and Supplementary Table 1 [file 41467_2020_15499_MOESM1_ESM.pdf]

Supplementary Information to

Prompt rewetting of drained peatlands reduces climate warming despite methane emissions

Anke Günther[1*], Alexandra Barthelmes[2,3], Vytas Huth[1], Hans Joosten[2,3], Gerald Jurasinski[1], Franziska Koebsch[1], John Couwenberg[2,3]

[1]University of Rostock, Faculty of Agricultural and Environmental Studies, Landscape Ecology, Rostock, Germany

[2]University of Greifswald, Faculty of Mathematics and Natural Sciences, Peatland Studies and Paleoecology, Greifswald, Germany

[3]Greifswald Mire Centre (GMC), Greifswald Germany

* Correspondence should be addressed to anke.guenther@uni-rostock.de

Supplementary Figures

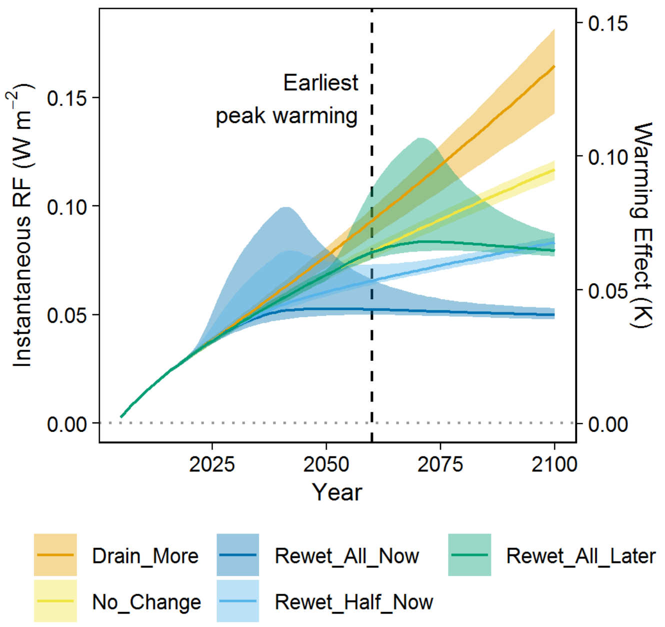

Supplementary Figure 1 Modeling sensitivity including methane peak. Sensitivity of radiative forcings (RF) and estimated warming effects of global peatland scenarios to modeling choices, uncertainty of emission factors and possibility of methane peaks after rewetting. Error ranges represent the range of radiative forcing resulting from random variations in ongoing drainage rate (1000-8000 km^2 per year), emission factors (10 % and 20 % uncertainty of emission factor), and occurrence of a methane peak (10 times rewetted methane emission factor for 5 years after rewetting).

Supplementary Tables

Supplementary Table 1 Assigned and aggregated emission factors (IPCC Wetlands Supplement) of Global Peatland Database (GPD) categories.

**GPD**		**IPCC**						
		**Field**				**Ditches**		
				**CO_2 from DOC**		**Cover fraction**	**CH_4**	
**Climatic zone**	**Land use category**	**Climatic zone**	**Emission factor**	**Climatic zone**	**Emission factor**		**Climatic zone**	**Emission factor**
Boreal	Forest	Boreal	Average of ‘Drained Forest Land Nutrient-poor’ and ‘Drained Forest Land Nutrient-rich’	Boreal	‘Drained’	2.5 %	Boreal / Temperate	‘Drained Forest Land / Drained Wetlands’
Boreal	Cropland	Boreal / Temperate	‘Drained Cropland’	Boreal	‘Drained’	5.0 %	Boreal / Temperate	‘Deep-drained Grassland / Cropland’
Boreal	Deep drained grassland	Boreal	‘Drained Grassland’	Boreal	‘Drained’	5.0 %	Boreal / Temperate	‘Deep-drained Grassland / Cropland’
Boreal	Shallow-drained grassland	-	-	-	-	-	-	-
Boreal	Agriculture	Boreal	Average of ‘Drained Cropland’ and ‘Drained Grassland’	Boreal	‘Drained’	5.0 %	Boreal / Temperate	Average of ‘Deep-drained Grassland / Cropland’ and ‘Shallow-drained Grassland’
Boreal	Peat extraction	Boreal / Temperate	‘Peatland Managed for Extraction’	Boreal	‘Drained’	5.0 %	Boreal / Temperate	‘Peat Extraction’
Boreal	Rewetted	Boreal	Average of ‘Rewetted Poor’ and ‘Rewetted Rich’	Boreal	‘Rewetted’	5.0 %	Boreal / Temperate	‘Drained Wetland / Forest’
Temperate	Forest	Temperate	‘Drained Forest Land’	Temperate	‘Drained’	2.5 %	Boreal / Temperate	‘Drained Forest Land / Drained Wetlands’
Temperate	Cropland	Boreal / Temperate	‘Drained Cropland’	Temperate	‘Drained’	5.0 %	Boreal / Temperate	‘Deep-drained Grassland / Cropland’
Temperate	Deep drained grassland	Temperate	Average of ‘Deep-drained Nutrient-rich Grassland’ and ‘Drained Nutrient-poor Grassland’	Temperate	‘Drained’	5.0 %	Boreal / Temperate	‘Deep-drained Grassland / Cropland’
Temperate	Shallow-drained grassland	Temperate	Average of ‘Shallow-drained Nutrient-rich Grassland’ and ‘Drained Nutrient-poor Grassland’	Temperate	‘Drained’	5.0 %	Boreal / Temperate	‘Shallow-drained Grassland’
Temperate	Agriculture	Temperate	Average of ‘Drained Cropland’,	Temperate	‘Drained’	5.0 %	Boreal /	Average of ‘Deep-drained

			‘Deep-drained Nutrient-rich Grassland’, ‘Shallow-drained Nutrient-rich Grassland’ and ‘Drained Nutrient-poor Grassland’				Temperate	Grassland / Cropland’ and ‘Shallow-drained Grassland’
Temperate	Peat extraction	Boreal / Temperate	‘Peatland Managed for Extraction’	Temperate	‘Drained’	5.0 %	Boreal / Temperate	‘Peat Extraction’
Temperate	Rewetted	Temperate	Average of ‘Rewetted Poor’ and ‘Rewetted Rich’	Temperate	‘Rewetted’	5.0 %	Boreal / Temperate	‘Drained Wetland / Forest’
Tropical	Forest	Tropical	‘Drained Forest Land’	Tropical	‘Drained’	2.0 %	Tropical	‘Drained’
Tropical	Cropland	Tropical	Average of ‘Drained Cropland and Fallow’ and ‘Drained Cropland: Paddy Rice’	Tropical	‘Drained’	2.0 %	Tropical	‘Drained’
Tropical	Deep drained grassland	Tropical	‘Drained Grassland’	Tropical	‘Drained’	2.0 %	Tropical	‘Drained’
Tropical	Shallow-drained grassland	-	-	-	-	-	-	-
Tropical	Agriculture	Tropical	Average of ‘Drained Cropland and Fallow’, ‘Drained Cropland: Paddy Rice’ and ‘Drained Grassland’	Tropical	‘Drained’	2.0 %	Tropical	‘Drained’
Tropical	Peat extraction	CO_2/N_2O: Tropical, CH_4: Boreal / Temperate	‘Peatland Managed for Extraction’	Tropical	‘Drained’	2.0 %	Tropical	‘Drained’
Tropical	Rewetted	Tropical	‘Rewetted’	Tropical	‘Rewetted’	2.0 %	Tropical	‘Drained’
