# Supplementary material for: Prompt rewetting of drained peatlands reduces climate warming despite methane emissions
Source: Nat Commun. 2020 Apr 2;11:1644. doi: 10.1038/s41467-020-15499-z (PMC7118086; doi:10.1038/s41467-020-15499-z)
Supplement: Supplementary file 2 — Supplementary Code [file 41467_2020_15499_MOESM2_ESM.docx]

Supplementary Code for

# Prompt rewetting of drained peatlands reduces climate warming despite methane emissions

Anke Günther^1*^, Alexandra Barthelmes^2,3^, Vytas Huth^1^, Hans Joosten^2,3^, Gerald Jurasinski^1^, Franziska Koebsch^1^, John Couwenberg^2,3^

^1^University of Rostock, Faculty of Agricultural and Environmental Studies, Landscape Ecology, Rostock, Germany

^2^University of Greifswald, Faculty of Mathematics and Natural Sciences, Peatland Studies and Paleoecology, Greifswald, Germany

^3^Greifswald Mire Centre (GMC), Greifswald Germany

* Correspondence should be addressed to [anke.guenther@uni-rostock.de](mailto:anke.guenther@uni-rostock.de)

## Supplementary Code

#####################################

### Atmospheric perturbation model for R ###

#####################################

### Model follows:

## Frolking et al. 2006 J. Geophys. Res.

## Neubauer & Megonigal 2015 Ecosystems

## Dommain et al. 2018 Global Change Biol.

## Joos et al. 2013 Atmos. Chem. Phys.

### Code by: Anke Guenther

## Version: 1.1

## Date: 2024-03-20

### The function requires a data frame similar to this for input

# data.frame(index = 1:100,

# co2flux = 0,

# ch4flux = 0,

# n2oflux = 0)

### The columns 'dataf$ch4flux', 'dataf$co2flux' and 'n2oflux' are required

### Fluxes at each time step have to be given in (g C /g N per time step)

### Time steps can be "month" or "year" and have to be specified (ts) in function

climmod <- function(dataf, ts){

### Setting starting values for function

## Fractions (a) and Lifetimes in years (tau) of each carbon pool

a0 <- 0.07

a1 <- 0.224

a2 <- 0.2824

a3 <- 0.2763

a4 <- 0.1473

tau0 <- 2e5

tau1 <- 394.4

tau2 <- 36.54

tau3 <- 4.304

tau4 <- 7000

## Atmospheric lifetimes for CH4-C and N2O-N

alt.ch4 <- 12.4

alt.n2o <- 121

# in years

# see Myhre et al. 2014

## Radiative efficiencies

radeff.co2 <- 1.756145e-15

radeff.ch4 <- 1.276547e-13

radeff.n2o <- 3.845573e-13

# in W m-2 kg-1

# see Myhre et al. 2014

## Indirect effects multiplier

ie.ch4 <- 1.65

ie.n2o <- 0.93

## Time steps

# Amount of time steps

ts.n <- nrow(dataf)

# Multiplier for lifetimes

ts.factor <- 1

if(ts == "month") {ts.factor <- 12}

### Calculate atmospheric inventories

## CO2-C

co2inv <- vector("numeric", length = ts.n) # CO2-C inventory in atmosphere

co2pool0 <- vector("numeric", length = ts.n) # Amount of CO2-C in pool 0

co2pool1 <- vector("numeric", length = ts.n) # Amount of CO2-C in pool 1

co2pool2 <- vector("numeric", length = ts.n) # Amount of CO2-C in pool 2

co2pool3 <- vector("numeric", length = ts.n) # Amount of CO2-C in pool 3

co2pool4 <- vector("numeric", length = ts.n) # Amount of CO2-C in pool 4

co2pool0[1] <- a0 * dataf$co2flux[1]

co2pool1[1] <- a1 * dataf$co2flux[1]

co2pool2[1] <- a2 * dataf$co2flux[1]

co2pool3[1] <- a3 * dataf$co2flux[1]

co2pool4[1] <- a4 * dataf$co2flux[1]

for(i in c(2:ts.n)){

co2pool0[i] <- a0 * dataf$co2flux[i] + co2pool0[i-1] * exp(-1/(ts.factor * tau0))

co2pool1[i] <- a1 * dataf$co2flux[i] + co2pool1[i-1] * exp(-1/(ts.factor * tau1))

co2pool2[i] <- a2 * dataf$co2flux[i] + co2pool2[i-1] * exp(-1/(ts.factor * tau2))

co2pool3[i] <- a3 * dataf$co2flux[i] + co2pool3[i-1] * exp(-1/(ts.factor * tau3))

co2pool4[i] <- a4 * dataf$co2flux[i] + co2pool4[i-1] * exp(-1/(ts.factor * tau4))

}

co2inv <- (co2pool0 +

co2pool1 +

co2pool2 +

co2pool3 +

co2pool4)

## CH4-C

# CH4-C inventory in atmosphere

ch4inv <- vector("numeric", length = ts.n)

ch4inv[1] <- dataf$ch4flux[1]

# CO2-C from CH4-C oxidation

ch4oxf <- vector("numeric", length = ts.n) # "flux" value: how much CH4-C is leaving the CH4-C pool by oxidation?

ch4oxpool0 <- vector("numeric", length = ts.n) # Amount of CO2-C from CH4-C oxidation in pool 0

ch4oxpool1 <- vector("numeric", length = ts.n) # Amount of CO2-C from CH4-C oxidation in pool 1

ch4oxpool2 <- vector("numeric", length = ts.n) # Amount of CO2-C from CH4-C oxidation in pool 2

ch4oxpool3 <- vector("numeric", length = ts.n) # Amount of CO2-C from CH4-C oxidation in pool 3

ch4oxpool4 <- vector("numeric", length = ts.n) # Amount of CO2-C from CH4-C oxidation in pool 4

ch4oxinv <- vector("numeric", length = ts.n) # Total amount of CO2-C from CH4-C oxidation

for(i in c(2:ts.n)){

if(ch4inv[i-1] > 0){ # Decay if the amount of CH4-C in atmosphere is positive

ch4inv[i] <- dataf$ch4flux[i] + ch4inv[i-1] * exp(-1/(ts.factor*alt.ch4))

# CH4-C oxidation

ch4oxf[i] <- ch4inv[i-1] * (1-exp(-1/(ts.factor*alt.ch4)))

ch4oxpool0[i] <- a0 * ch4oxf[i] + ch4oxpool0[i-1] * exp(-1/(ts.factor*tau0))

ch4oxpool1[i] <- a1 * ch4oxf[i] + ch4oxpool1[i-1] * exp(-1/(ts.factor*tau1))

ch4oxpool2[i] <- a2 * ch4oxf[i] + ch4oxpool2[i-1] * exp(-1/(ts.factor*tau2))

ch4oxpool3[i] <- a3 * ch4oxf[i] + ch4oxpool3[i-1] * exp(-1/(ts.factor*tau3))

ch4oxpool4[i] <- a4 * ch4oxf[i] + ch4oxpool4[i-1] * exp(-1/(ts.factor*tau4))

}

else { # No decay if there has been net uptake from the atmosphere

ch4inv[i] <- dataf$ch4flux[i] + ch4inv[i-1]

# CO2-C from previous CH4-C oxidation continues to equlibrate with C pools

ch4oxpool0[i] <- ch4oxpool0[i-1] * exp(-1/(ts.factor*tau0))

ch4oxpool1[i] <- ch4oxpool1[i-1] * exp(-1/(ts.factor*tau1))

ch4oxpool2[i] <- ch4oxpool2[i-1] * exp(-1/(ts.factor*tau2))

ch4oxpool3[i] <- ch4oxpool3[i-1] * exp(-1/(ts.factor*tau3))

ch4oxpool4[i] <- ch4oxpool4[i-1] * exp(-1/(ts.factor*tau4))

}}

ch4oxinv <- (ch4oxpool0 +

ch4oxpool1 +

ch4oxpool2 +

ch4oxpool3 +

ch4oxpool4)

## N2O-N

n2oinv <- vector("numeric", length = ts.n) # N2O-N inventory in atmosphere

n2oinv[1] <- dataf$n2oflux[1]

for(i in c(2:ts.n)){

if(n2oinv[i-1] > 0){ # Decay if the amount of N2O-N in atmosphere is positive

n2oinv[i] <- dataf$n2oflux[i] + n2oinv[i-1] * exp(-1/(ts.factor*alt.n2o))}

else { # No decay if there has been net uptake from the atmosphere

n2oinv[i] <- dataf$n2oflux[i] + n2oinv[i-1]

}}

## Convert all inventories to kg,

## then calculate individual instantaneous radiative forcing

dataf$n2orf <- (n2oinv /1000 /28 *44) * radeff.n2o * ie.n2o

dataf$ch4rf <- (ch4inv /1000 /12 *16) * radeff.ch4 * ie.ch4

# RF of CH4 in atmosphere

dataf$ch4oxrf <- (ch4oxinv /1000 /12 *44) * radeff.co2

# RF of CO2 from CH4 oxidation

dataf$ch4totrf <- dataf$ch4rf + dataf$ch4oxrf

# Combined RF of CH4 and CO2 from CH4 oxidation

dataf$co2rf <- (co2inv /1000 /12 *44)* radeff.co2

## Calculate total radiative forcing

dataf$totalforcing <- dataf$n2orf + dataf$ch4totrf + dataf$co2rf

# Through total GHGs present in atmosphere at each time step

return(dataf)

}
